# Eye-tracking during newborn intubations

**DOI:** 10.1038/s41390-023-02630-1

**Published:** 2023-04-28

**Authors:** Philipp K. Buehler, Pedro David Wendel-Garcia, Daniel A. Hofmaenner

**Affiliations:** Institute of Intensive Care Medicine, University Hospital Zurich, Zurich, Switzerland

With great interest, we read the recently published article by Kessler et al. in the Journal.^1^ In a well-designed study, the authors present some novel insights into the visual behavior of healthcare professionals while performing neonatal intubations.^1^ We would like to highlight some points of interest that are worth discussing.

First, eye-tracking research is relatively novel in the field of intensive care medicine and research has so far often been restricted to limited participant numbers. Recently, it has been used more frequently to assess visual behavior in the critical care environment.^2^ The authors should thus be congratulated for conducting their study in a multi-centric design, adding external validity to their findings. However, the authors conducted their trial in a simulated setting.^1^ This choice of study design unfortunately precludes observation of a lot of the confounding effects existent in a real-life scenario, which eye-tracking enables to crystalize. Actions performed in emergency situations are commonly associated with more stress and insecurity as well as with more interactions with other healthcare providers and monitoring equipment, than simulated cases. The nuances of eye-tracking really shine out in these more complex environments, as interfering events can actively be identified.

Previous research from our own group has demonstrated that eye-tracking is feasible in real-life scenarios in the intensive care unit, including during the investigation of high-risk interventions. For instance, the technology of eye-tracking was successfully used during the visual assessment of extubations of real, critically ill patients, where it could uncover clear mismatches in perceived versus real patient assessment.^3^ Undoubtedly, real-life intubations of newborns would thus enhance our understanding of visual awareness of healthcare personnel and add even more generalizability to the author’s findings. Of note, real-life studies in critically ill newborns have been successfully performed.^4^

Second, while the study of Kessler et al. analyzed the number of fixations and dwell time on their pre-specified areas of interest (AOIs), the inclusion of the revisit ratio would probably have provided further helpful insights into their analysis. The revisit ratio can be interpreted as a surrogate of the complexity of a particular AOI. Moreover, it is known to be indicative of visual patterns related to checks and controlling, and thus of increased neurocognitive focus.^5^ It would have been interesting to elucidate whether revisits on distinct AOIs would have differed among participants with different experience levels.

Third, in order to synthesize faithful information of using eye-tracking, attention has to be paid in order to keep the participant cohorts homogeneous, as the sample size cannot be indefinitely increased at the moment due to time constraints in the evaluation of videos. The large heterogeneity in professional positions, age and experience observed in this trial might thus have restricted the observation of more nuanced signals in the data.^1^ Finally, we would recommend to consider the integration of individual stress levels of participants into the analysis. It is plausible that visual behavior can be significantly affected by physical or emotional stress.

With much interest, we are awaiting further eye-tracking trials assessing high-risk situations in the critical care environment. Undoubtedly, novel insights could be gained that might assist to improve patient outcomes in the future.
